# Supplementary material for: The mitochondrial DNA polymerase gamma degrades linear DNA fragments precluding the formation of deletions
Source: Nat Commun. 2018 Jun 27;9:2491. doi: 10.1038/s41467-018-04895-1 (PMC6021392; doi:10.1038/s41467-018-04895-1)
Supplement: Supplementary file 1 — Supplementary Information [file 41467_2018_4895_MOESM1_ESM.pdf]

The Mitochondrial DNA Polymerase Gamma Degrades Linear DNA Fragments Precluding the Formation of Deletions

Nissanka et al.

SUPPLEMENTARY FIGURES

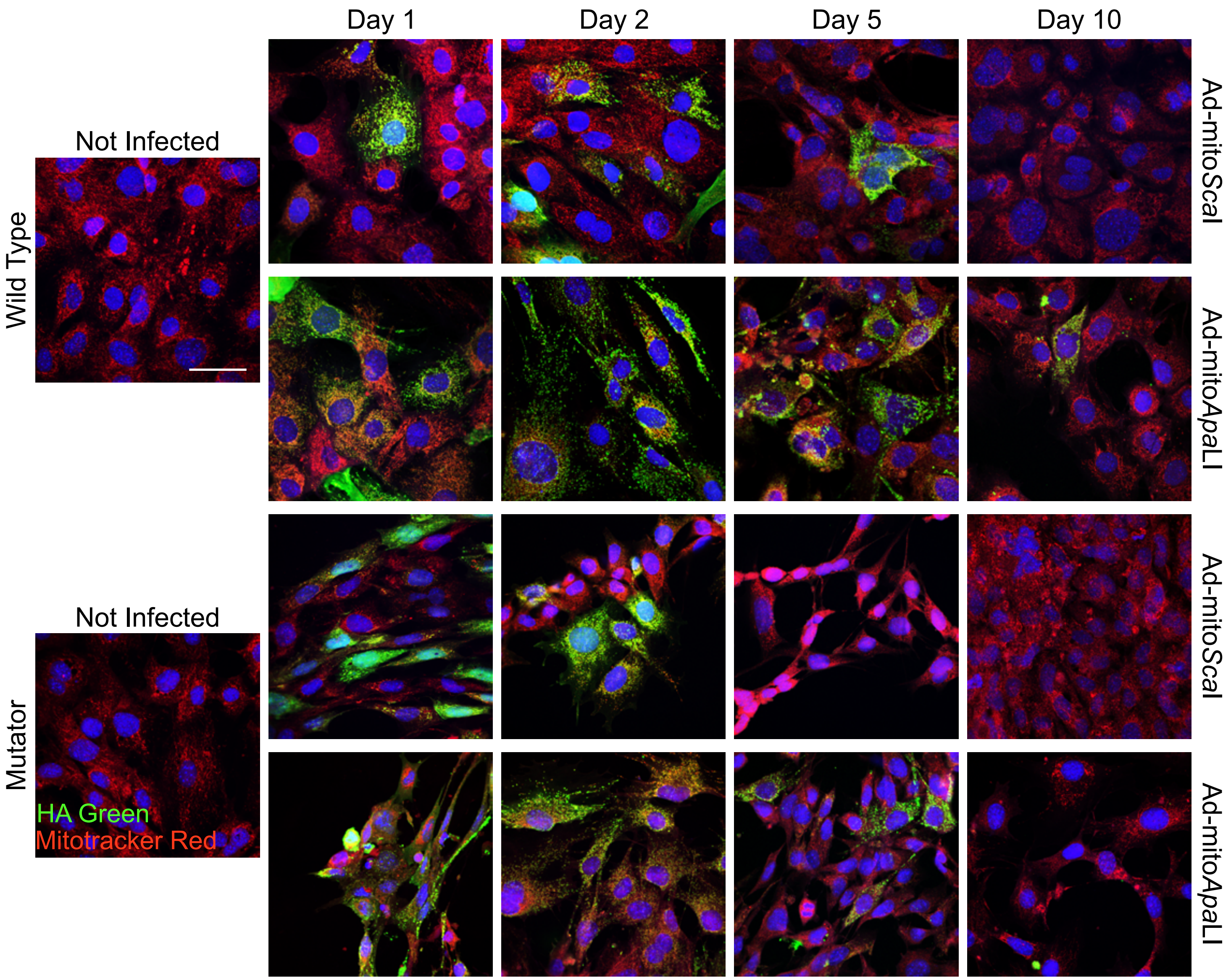

Supplementary Figure 1. Expression of mitochondrial-targeted restriction endonucleases in fibro-blasts of the mutator mouse. This figure expands on figure 1 by including days 1 and 5. Lung fibro-blasts from wild-type and mutator mice were infected with adenovirus expressing mito*Sca*I-HA or mito*Apa*LI-HA restriction endonucleases. Cells were stained with Mitotracker Red and fixed after 1, 2, 5 and 10 days. Cells were immuno-stained for HA. Scale bar = 50 μm.

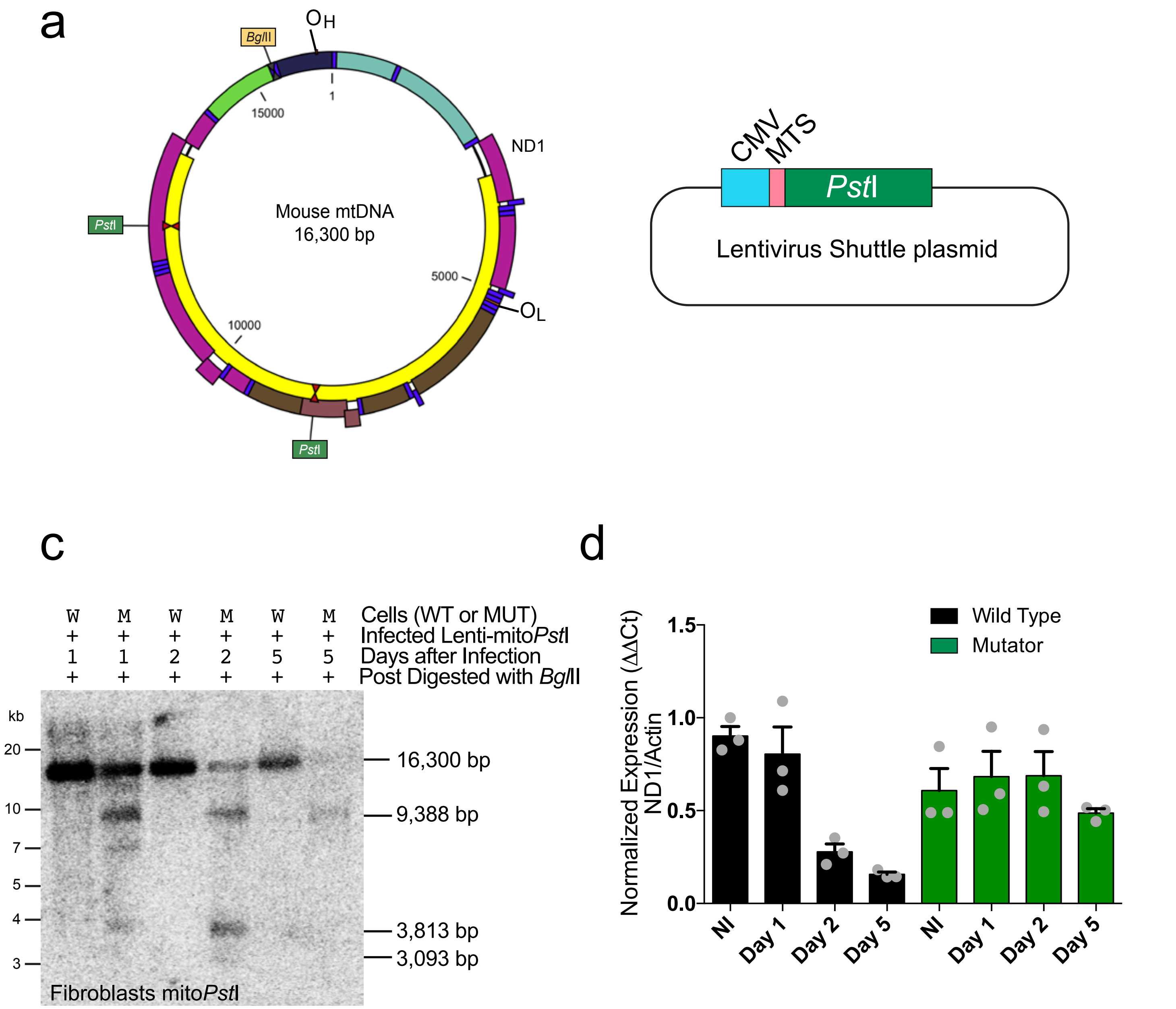

Supplementary Figure 2. MtDNA after mitochondrial-targeted PstI in wild-type and mutator fibroblasts. a) Diagram of wild-type C57BL6/J mitochondrial DNA illustrating the location and number of *Pst*I restriction endonuclease sites. b) Diagram of the plasmid used to make lentivirus expressing mito*Pst*I. c) Southern blot from mutator and wild-type cells infected with lenti-mito*Pst*I 1, 2, and 5 days post-infection. Purified DNA was post-digested with *Bgl*II to linearize mitochondrial DNA. Zeta-probe membranes were hybridized with a [α-^{32}P] dCTP labeled probe covering 11.7 kb of mitochondrial DNA. d) Relative quantity of total mitochondrial DNA levels were assessed by qPCR using ND1 (mitochondrial DNA) and β-Actin (genomic DNA) primers in DNA from mutator and wild-type fibroblasts infected with lenti-mito*Pst*I at 1, 2, and 5 days post-infection. Quantification was done using comparative Ct method. Error bars (s.e.m) derive from technical replicates.

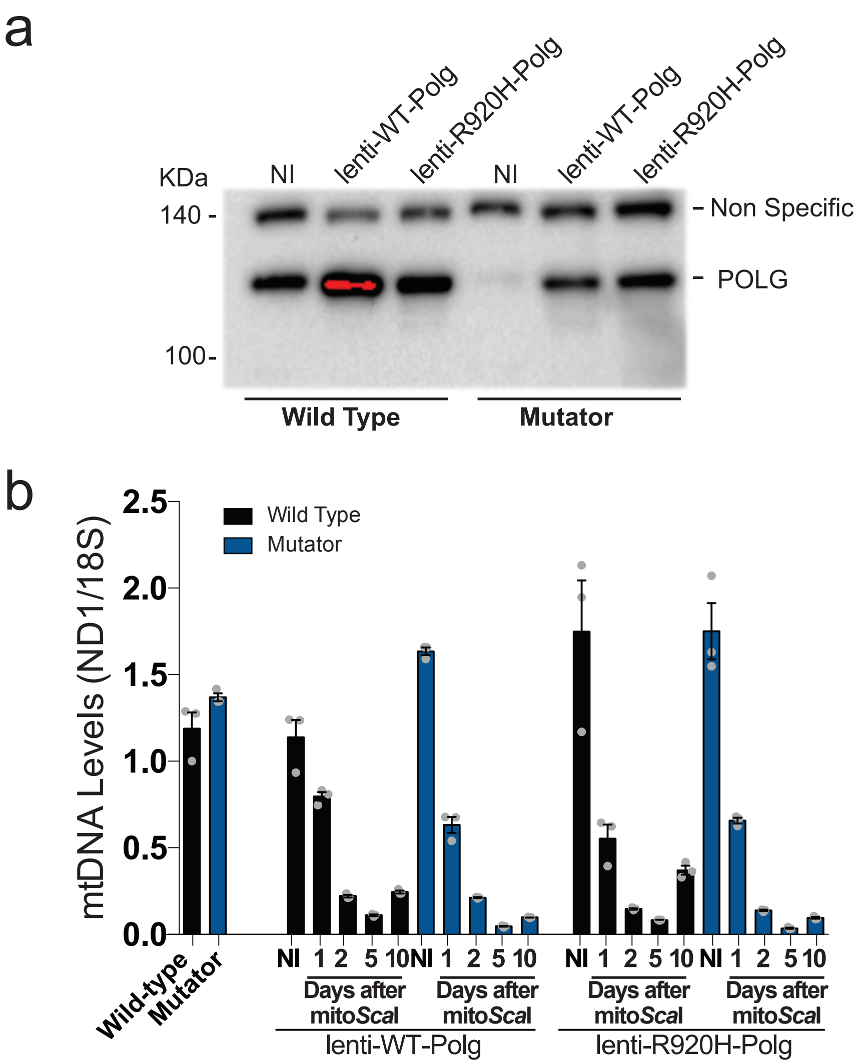

Supplementary Figure 3. MtDNA levels after DSB following expression of POLG without polymerase activity in mutator mouse cells. Wild-type and mutator fibroblasts were infected with lentiviruses encoding for either the wild-type exonuclease, wild-type polymerase or wild-type exonuclease, R920H polymerase mouse polymerase gamma A subunit. a) Expression of the different recombinant lentivirus using a POLG antibody (Abcam #ab128899). b) Effect of these expressions on mtDNA levels. Five days after recombinant lentiviral expression, double-strand breaks were induced with adenovirus encoding for Ad-mito*Sca*I-HA. Mutator and wild-type fibroblasts infected with Ad-mito*Sca*I-HA at 1, 2, 5, and 10 days post-Polg lentiviral infection. Relative levels of total mtDNA were assessed using ND1 (mtDNA) and 18S (nuclear DNA) primers (normalized ΔΔCt). Error bars (s.e.m.) derive from technical replicates.

a — Deleted Region

b — Number of occurences

a: Breakpoint sequence	5' position	Deletion size	3' position	WT Fibro	MUT Fibro	WT Liver	MUT Liver
CTCTCCAAACCCCCCACCCCCTCCTCTTAATGCCA...AGTACTTCTAGCATCAGGTGTTTCAATTACATGAGCTCATCATAGCC	16,099	9,243 bp	9,042	2			3
CTCTCCAAACCCCCCACCCCCTCCTCTTAATGCCA...AGTACTTCTAGCATCAGGTGTTTCAATTACATGAGCTC	16,099	9.234 bp	9,033				2
CTCCAAACCCCCCACCCCCTCCTCTTAATGCCAAA...GAGCTCATCATAGCCTTATAGAAGGTAAACGAAAC	16,101	9,261 bp	9,062				1
AACCCCCCACCCCCTCCTCTTAATGCCAAACCCCA...AGTACTTCTAGCATCAGGTGTTTCAATTACATGAGCTCATCATAGCCTTATAGAAGGT	16,106	9,247 bp	9,053		1		
CCCACCCCCTCCTCTTAATGCCAAACCCCAAAAAC...ATCATAGCCTTATAGAAGGTAAACGAAACCACATA	16,111	9,257 bp	9,068		1		
CTCTTAATGCCAAACCCCAAAAACACTAAGAACTT...CCTTATAGAAGGTAAACGAAACCACATAAATCAAG	16,122	9,253 bp	9,075		1		
TAATGCCAAACCCCAAAAACACTAAGAACTTGAAA...AGTACTTCTAGCATCAGGTGTTTCAATTACATGAGCTCATCATAGCCTTAT	16,126	9,220 bp	9,046			1	
ATGCCAAACCCCAAAAACACTAAGAACTTGAAAGA...AGTACTTCTAGCATCAGGTGTTTCAATTACATGAGCTCATCATAGCCTTATAGA	16,128	9,221 bp	9,049	1			
TGCCAAACCCCAAAAACACTAAGAACTTGAAAGAC...CCTTATAGAAGGTAAACGAAACCACATAAATCAAG	16,129	9,246 bp	9,075			1	
GCCAAACCCCAAAAACACTAAGAACTTGAAAGACA...AGTACTTCTAGCATCAGGTGTTTCAATTACATGAGCTCATCATAGCCT	16,130	9,213 bp	9,043				1
CCAAAAACACTAAGAACTTGAAAGACATATAATAT...AGTACTTCTAGCATCAGGTGTTTCAATTACATGAGCTCATCATAGCC	16,138	9,204 bp	9,042			1	
CTAAGAACTTGAAAGACATATAATATTAACTATCA...GAGCTCATCATAGCCTTATAGAAGGTAAACGAAAC	16,147	9,215 bp	9,062		1		
ATATAATATTAACTATCAAACCCTATGTCCTGATC...TGAGCTCATCATAGCCTTATAGAAGGTAAACGAAA	16,164	9,197 bp	9,061			1	
TTAACTATCAAACCCTATGTCCTGATCAATTCTAG...CCTTATAGAAGGTAAACGAAACCACATAAATCAAG	16,172	9,203 bp	9,075				1
ACCCTATGTCCTGATCAATTCTAGTAGTTCCCAAA...AGTACTTCTAGCATCAGGTGTTTCAATTACATGAGCTCATCATAGCCTTATAGA	16,183	9,166 bp	9,049			1	
ACCCTATGTCCTGATCAATTCTAGTAGTTCCCAAA...AGTACTTCTAGCATCAGGTGTTTCAAT	16,183	9,139 bp	9,022			1	
TGATCAATTCTAGTAGTTCCCAAAATATGACTTATATTTTAGTACT...ATAGCCTTATAGAAGGTAAACGAAACCACATAAAT	16,194	9,177 bp	9,071			1	
ATCAATTCTAGTAGTTCCCAAAATATGACTTATATTTTAGTACT...CCTTATAGAAGGTAAACGAAACCACATAAATCAAG	16,196	9,179 bp	9,075		4		
TCAATTCTAGTAGTTCCCAAAATATGACTTATATTTTAGTACT...AGTACTTCTAGCATCAGGTGTTTCAATTACATGAGCTCATC	16,197	9,139 bp	9,036	1			
CAATTCTAGTAGTTCCCAAAATATGACTTATATTTTAGTACT...AGTACTTCTAGCATCAGGTGTTTCAATTACATGAGCT	16,198	9,134 bp	9,032		1		
TAGTAGTTCCCAAAATATGACTTATATTTTAGTACT...AGTACTTCTAGCATCAGGTGTTTCAATTACATGAGCTCATCATAGCCT	16,204	9,139 bp	9,043	1			
TAGTAGTTCCCAAAATATGACTTATATTTTAGTACT...CCACTACTTAATACTTCAGTACTTCTAGCATCAGG	16,204	9,109 bp	9,013		1		
GTTCCCAAAATATGACTTATATTTTAGTACT...AGTACTTCTAGCATCAGGTGTTTCAATTAC	16,209	9,116 bp	9,025			1	

Supplementary Figure 4. Sequence analyses of mtDNA deletion breakpoints from the breakpoint [with O_H]. PCR amplifications from DNA of fibroblasts and liver infected with Ad-mito*Sca*I-HA (wild-type and mutator) were cloned in a TOPO TA plasmid (TA cloning) and sequenced using M13F and M13R primers. a) 3' and 5' breakpoints are annotated as follows, orange text - mtDNA that is retained, black text -mtDNA which is deleted, blue text - *Sca*I sequence, green text - size of the deletion breakpoint, underlined text - micro or imperfect homologies b) Table of breakpoint frequencies and sample origin of the breakpoint.

a — Deleted Region | **b** — Number of occurences

Breakpoint sequence	5' position	Deletion size	3' position	WT Fibro	MUT Fibro	WT Liver	MUT Liver
CGTAATTCGTGAAGGAACCTACCAAGGCCACCACA...ACAGCATTTATAGGCTACGTCCTTCCATGAGGACA	8,801	5,739 bp	14,539	1			
TACCAAGGCCACCACACTCCTATTGTACAAAAAGG...ACAAATATCATTCTGAGGTGCCACAGTTATTACAA	8,820	5,752 bp	14,571		1		
TATTCATCGTCTCGGAAGTATTTTTCTTTGCAGGA...GGTGCCACAGTTATTACAAACCTCCTATCAGCCAT	8,875	5,713 bp	14,587		1		
TCATCGTCTCGGAAGTATTTTTCTTTGCAGGATTC...AGTACTTCTACTGTTCGCAGTCATAGCCACAGCATTTATAGGCTACGTCCTTCCA	8,878	5,654 bp	14,531		1		
AAGTATTTTTCTTTGCAGGATTCTTCTGAGCGTTC...AGTACTTCTACTGTTCGCAGTCATAGCCACAGCATTTATAGGCTACGTCCTTCCA	8,890	5,642	14,531		1		
TTCTTTGCAGGATTCTTCTGAGCGTTCTATCAT...AGTACTTCTACTGTTCGCAGTCATAGCCACAGCATTTATAGGCTACGTCCTTCCATGAGG	8,896	5,643 bp	14,538		1		
GCAGGATTCTTCTGAGCGTTCTATCATTCTAGCCT...TGGAACAACCCTAGTCGAATGAATTTGAGGGGGCT	8,904	5,728 bp	14,631	1			
CTGAGCGTTCTATCATTCTAGCCTCGTACCAACAC...ATGAGGACAAATATCATTCTGAGGTGCCACAGTTA	8,915	5,651 bp	14,565	1			
TCTATCATTCTAGCCTCGTACCAACACATGATCTA...AGTACTTCTACTGTTCGCAGTCATAGCCACAGCATTTATAG	8,923	5,595 bp	14,517		1		
TCTAGCCTCGTACCAACACATGATCTAGGAGGCTG...AGTACTTCTACTGTTCGCAGTCATAGCCACAGCATTTATAG	8,931	5,587 bp	14,517				1
GATCTAGGAGGCTGCTGACCTCCAACAGGAATTTC...AGTACTTCTACTGTTCGCAGTCATAGCCACAGCATTTATAGG	8,952	5,567 bp	14,518	1			
TCTAGGAGGCTGCTGACCTCCAACAGGAATTTCAC...AGTACTTCTACTGTTCGCAGTCATAGCCACAGCATTTA	8,954	5,561 bp	14,514		1		
CTAGGAGGCTGCTGACCTCCAACAGGAATTTCACC...CCATGAGGACAAATATCATTCTGAGGTGCCACAGT	8,955	5,609 bp	14,563				1
AGGAGGCTGCTGACCTCCAACAGGAATTTCACCAC...TGCCACAGTTATTACAAACCTCCTATCAGCCATCC	8,957	5,633 bp	14,589				1
AGGAGGCTGCTGACCTCCAACAGGAATTTCACCAC...CCTTCCATGAGGACAAATATCATTCTGAGGTGCCA	8,957	5,603 bp	14,559				1
GAGGCTGCTGACCTCCAACAGGAATTTCACCACTT...TCCATGAGGACAAATATCATTCTGAGGTGCCACAG	8,959	5,604 bp	14,562	1			
AGGCTGCTGACCTCCAACAGGAATTTCACCACTTA...AGTACTTCTACTGTTCGCAGTCATAGCCACAGCATTTATAG	8,960	5,558 bp	14,517				1
GACCTCCAACAGGAATTTCACCACTTAACCCTCTA...AGTACTTCTACTGTTCGCAGTCATAGCCACAGCATTTAT	8,968	5,548 bp	14,515	1			
ACCTCCAACAGGAATTTCACCACTTAACCCTCTAG...AGTACTTCTACTGTTCGCAGTCATAGCCACAGCATTTATAG	8,969	5,549 bp	14,517		1		
AACAGGAATTTCACCACTTAACCCTCTAGAAGTCCCACTACTTAATACTTCAGTACT...ATGAGGACAAATATCATTCTGAGGTGCCACAGTTA	8,973	5,593 bp	14,565	2			
CAGGAATTTCACCACTTAACCCTCTAGAAGTCCCACTACTTAATACTTCAGTACT...AGGTGCCACAGTTATTACAAACCTCCTATCAGCCA	8,977	5,610 bp	14,586				1
CCACTTAACCCTCTAGAAGTCCCACTACTTAATACTTCAGTACT...ATAGCCACAGCATTTATAGGCTACGTCCTTCCATG	8,988	5,546 bp	14,533		1		
TTAACCCTCTAGAAGTCCCACTACTTAATACTTCAGTACT...CTGAAACATTGGAGTACTTCTACTGTTCGC	8,992	5,503 bp	14,494				1
CTAGAAGTCCCACTACTTAATACTTCAGTACT...AGTACTTCTACTGTTCGCAGTCATAGCCACAGCATTTA	9,000	5,515 bp	14,514				1
CTAGAAGTCCCACTACTTAATACTTCAGTACT...GTTATTACAAACCTCCTATCAGCCATCCCATATAT	9,000	5,597 bp	14,596				1
AGAAGTCCCACTACTTAATACTTCAGTACT...CCTGAAACATTGGAGTACTTCTACTGTTCG	9,002	5,492 bp	14,493	1			
ACTTAATACTTCAGTACTTCTA...AGTACTTCTACTGTTCGCAGTCATA	9,014	5,488 bp	14,501				1
TAATACTTCAGTACTTCTAGCATCAGGTGT...AACATTGGAGTACTTCTACTGTTCGCAGT	9,017	5,482 bp	14,498			1	
ACTTCAGTACTTCTAGCATCAGGTGTTTCA...AACCTGAAACATTGGAGTACTTCTACTGTT	9,021	5,471 bp	14,491			2	

Supplementary Figure 5. Sequence analyses of mtDNA deletion breakpoints from the breakpoint [with O_H and O_L]. PCR amplifications from DNA of fibroblasts and liver infected with Ad-mito*Sca*I-HA (wild-type and mutator) were cloned in a TOPO TA plasmid (TA cloning) and sequenced using M13F and M13R primers. a) 3' and 5' breakpoints are annotated as follows, orange text - mtDNA that is retained, black text -mtDNA which is deleted, blue text - *Sca*I sequence, green text - size of the deletion breakpoint, underlined text - micro or imperfect homologies b) Table of breakpoint frequencies and sample origin of the breakpoint.
